# Translator from Extended SysML to Physical Interaction and Signal Flow Simulation Platforms, Version 1.1

**DOI:** 10.6028/jres.126.027

**Published:** 2021-09-22

**Authors:** Raphael Barbau, Conrad Bock, Mehdi Dadfarnia

**Affiliations:** 1 Engisis LLC, Bethesda, MD 20817, USA; 2 Associate, National Institute of Standards and Technology, Gaithersburg, MD, USA; 3 National Institute of Standards and Technology, Gaithersburg, MD, USA

**Keywords:** lumped-parameter simulation, model-based systems engineering, model transformation, SysML

**Software DOI:** https://doi.org/10.18434/mds2-2414

## 1 Summary

The design of complex systems often requires engineers from multiple disciplines (mechanical, electrical, production, and so on) to communicate with each other and exchange system design information. Systems engineering models are a cross-disciplinary foundation for this process, but are not well-integrated with specialized engineering information, leading to redundant and inconsistent system specifications. The software provided here translates system models in the Systems Modeling Language (SysML) [1] to physical interaction and signal flow (also known as lumped-parameter, one-dimensional, or network) models on two simulation platforms used in many engineering domains.

The translator implements the SysML Extension for Physical Interaction and Signal Flow Simulation (SysPhS) version 1.1, published by the Object Management Group [2]. It can generate Modelica^1^1Certain commercial equipment, instruments, materials, or software are identified in this paper to specify the experimental procedure adequately. Such identification is not intended to imply recommendation or endorsement by the National Institute of Standards and Technology, nor is it intended to imply that the materials or equipment identified are necessarily the best available for the purpose. or Simulink/Simscape files from SysML models extended according to the SysPhS specification. This translator is an updated version of the SysPhS 1.0 translator [3].

Several example models are provided to demonstrate the translator in various engineering domains.

## 2 Software Specifications

**Table:** 

**NIST Operating Unit**	Engineering Laboratory, Systems Integration Division
**Category**	System model translator
**Targeted Users**	Systems engineers, simulation engineers
**Operating Systems**	See systems requirements of Java 8 or later
**Programming Language**	Java 8
**Inputs/Outputs**	Input: SysML models created using the SysPhS profile Output: Modelica or Simulink file corresponding to the model
**Documentation**	Instructions on how to run the translator are provided in the software package, see README.txt file. The translator is an implementation of the SysPhS specification, available at https://www.omg.org/spec/SysPhS/1.1. The source code of the translator is available at https://doi.org/10.18434/mds2-2414
**Accessibility**	N/A
**Disclaimer**	https://www.nist.gov/director/licensing

## 3 Software details

The software translates SysML models extended with SysPhS into simulation files for Simulink/Simscape and Modelica, per the SysPhS specification. The SysML models must be serialized in XML Metadata Interchange file format (XMI) [4]. The software deserializes these files into instances of the SysML metamodel using the Eclipse Modeling Framework (EMF) library and its extension for the Unified Modeling Language (UML) [5], on which SysML is based. EMF was used to create metamodels of the Simulink/Simscape and Modelica languages. Java libraries were automatically generated from these metamodels. These libraries are then used to instantiate the metamodels based on the input SysML models. The result is then serialized into simulation files that can be opened by their simulation platforms. Additional explanation is available in [6, 7].

The translator also supports the reverse translation, from Simulink/Simscape and Modelica files generated by the translator to extended SysML.

## 4 Methods for Validation

A set of SysML example models are included to demonstrate the translator. These sample models cover multiple aspects of SysPhS:

⏺ An electrical circuit model demonstrates how to use physical interaction modeling in the electrical domain.
⏺ A hydraulics model demonstrates how to use physical interaction modeling in the hydraulic domain.
⏺ A signal processor model (with low-pass and high-pass filter) demonstrates how to use signal-flow modeling.
⏺ A humidifier system model demonstrates how to use signal-flow modeling to describe the operation of a humidifier in a room. The model also includes simple state machines.
⏺ A cruise controller model demonstrates mixed usage of physical interaction and signal-flow. The signal flow portion covers speed sensors and engine actuators, while the physical interaction portion covers the flow of mechanical energy that powers the car and makes it move.

The sample models also include another version of the cruise controller model, which is used in a publication on debugging physical interaction and signal for models [8].

All these models were translated into Modelica and Simulink/Simscape files that simulate successfully, showing how the systems will behave over time.
